# Exosomal MicroRNA-155 Inhibits Enterovirus A71 Infection by Targeting PICALM

**DOI:** 10.7150/ijbs.36388

**Published:** 2019-11-15

**Authors:** Jing Wu, Jiaqi Gu, Li Shen, Daihua Fang, Xinran Zou, Yuwen Cao, Shengjun Wang, Lingxiang Mao

**Affiliations:** 1 Department of Laboratory Medicine, The Affiliated People's Hospital, Jiangsu University, Zhenjiang, China; 2 Department of Immunology, Jiangsu Key Laboratory of Laboratory Medicine, School of Medicine, Jiangsu University, Zhenjiang, China; 3 Clinical Laboratory, Zhenjiang Center for Disease Control and Prevention, Jiangsu, China; 4 Clinical Laboratory, Xuzhou Children's Hospital, Xuzhou, China

**Keywords:** Enterovirus A71, Exosomes, MicroRNA-155, PICALM

## Abstract

Enterovirus A71 (EV-A71) causes hand, foot, and mouth disease (HFMD) that is associated with neurological complications. Researchers have shown that exosomes containing host cellular microRNA (miRNA) can modulate the recipient's cellular response during viral infection. However, it is unclear how exosomal miRNAs regulate this response during EV-A71 infection. In this study, we used an exosomal miRNA chip to show that microRNA-155 (miR-155) was markedly enriched in exosomes after EV-A71 infection. Moreover, exosomal miR-155 efficaciously inhibited EV-A71 infection by targeting phosphatidylinositol clathrin assembly protein (PICALM) in recipient cells. Importantly, we confirmed that exosomal miR-155 reduced EV-A71 infection severity *in vivo*. Additionally, miR-155 levels in throat swabs from EV-A71-infected patients were higher than in those from healthy individuals. Collectively, our findings provide evidence that exosomal miR-155 plays a role in host-pathogen interactions by mediating EV-A71 infection via the repression of PICALM; these results provide insights into the regulatory mechanisms of viral infection.

## Introduction

Enterovirus A71 (EV-A71) is one of the main pathogenic factors of hand, foot, and mouth disease (HFMD). These infections are usually mild and self-limiting, but severe HFMD caused by EV-A71 leads to neurological complications 1. Elucidation of the cellular events following EV-A71 infection will facilitate the development of strategies to prevent and treat viral infection. However, the molecular mechanisms of the host response to EV-A71 infection are not completely understood.

MicroRNAs (miRNAs), a class of endogenous, non-coding RNAs, combine with the 3′untranslated region (3′UTR) or coding region of the target mRNA to degrade target mRNA or inhibit the translation of target genes 2. Previous studies showed that viral infection can be modulated by host-derived miRNAs such as miR-122 and miR-29a 3, 4. Exosomes are bioactive extracellular nanovesicles of approximately 30-150 nm that are secreted by various cell types and are widely distributed in body fluids 5. Exosomes can deliver functional miRNAs, proteins, and lipids to recipient cells and therefore play an important role in many physiological or pathological processes, particularly infectious diseases 6, 7. Exosomes can shuttle both infectious cargo and protective host molecules between cells. For instance, the hepatitis C virus (HCV) genome can be packaged in exosomes and excreted out of the cells to generate an effective infection 8, 9. In addition, some antiviral molecules (hsa-miR-1260, hsa-miR-4284, hsa-miR-638, etc.) can be delivered from liver non-parenchymal cells to hepatocytes via exosomes 10. Nonetheless, the physiological significance of exosomes in the delivery of bioactive molecules to the host defense system is worthy of further investigation.

In this study, we demonstrate that up-regulated miR-155 is directly transferred from infected cells to uninfected cells through exosomes. Exosomal miR-155 can inhibit EV-A71 infection by down-regulating phosphatidylinositol clathrin assembly protein (PICALM) *in vitro* and *in vivo*. Importantly, our findings show the antiviral activity of exosomal miR-155 and identify miR-155 as a potential therapeutic agent against severe EV-A71 infection.

## Results

### Exosomal miR-155 inhibits EV-A71 infection *in vitro*

**Figure 1:**
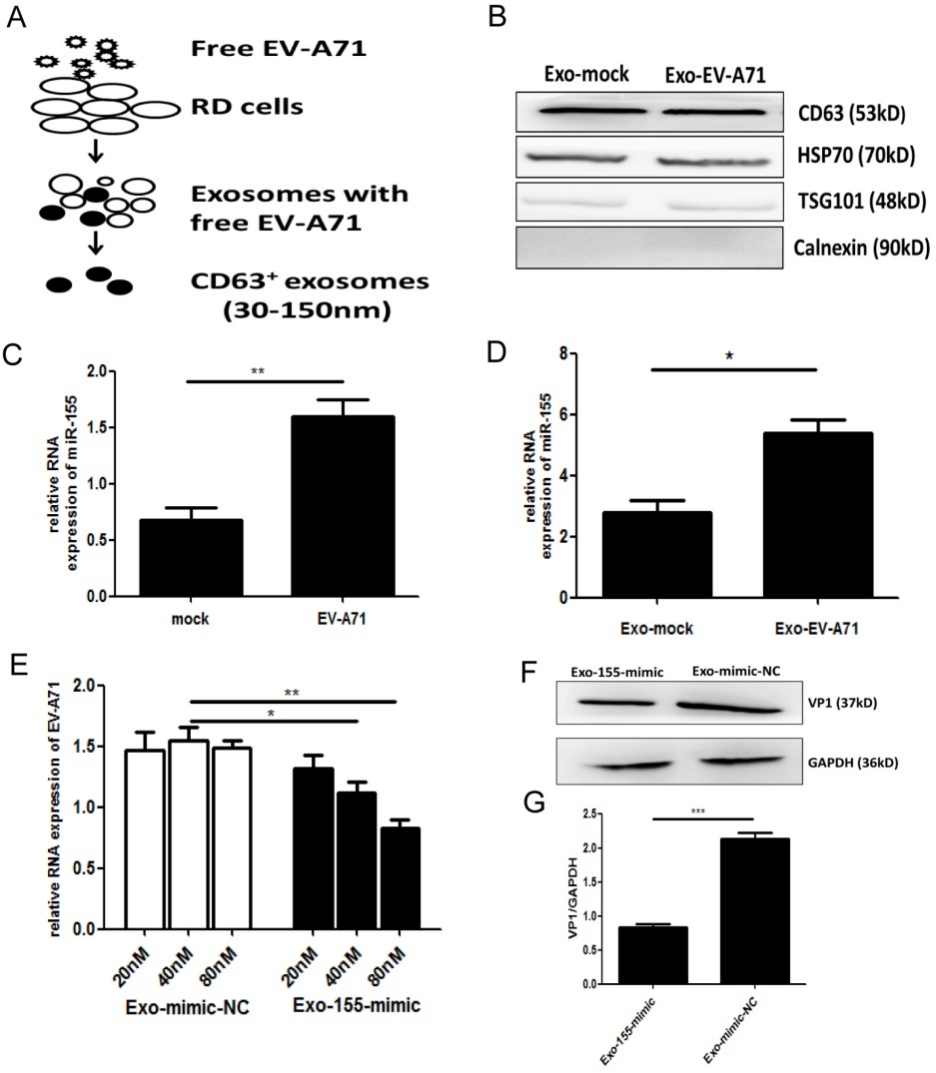
** Exosomal miR-155 inhibits EV-A71 infection *in vitro*. (A)** A two-step approach was used to isolate exosomes from the supernatants of EV-A71-infected or uninfected RD cells. **(B)** Exosomal marker proteins: tetraspanin CD63, heat shock protein 70 (HSP70), tumor suppressor gene 101 protein (TSG101). The endoplasmic reticulum marker calnexin was absent from the exosomes. **(C)** qRT-PCR analysis of miR-155 RNA levels in EV-A71-infected RD cells. U6 small nuclear RNA (U6snRNA) was used as an internal control for miRNA detection. **(D)** qRT-PCR analysis of the miR-155 RNA level in exosomes at 36 h p.i. Cel-miR-39 was used as an internal control for exosomal miRNA detection. Viral genomic RNA **(E)** and VP1 protein **(F)** were detected at 36 h p.i. after incubation with exosomal miR-155-mimic (Exo-155-mimic) or miR-mimic-NC (Exo-mimic-NC). **(G)** Normalization of VP1 protein with endogenous protein glyceraldehyde 3-phosphate hydrogenase (GAPDH) was carried out with ImageJ software (National Institutes of Health, USA). Data are from three independent experiments with each experiment performed in triplicate. *p < 0.05, **p < 0.01, ***p < 0.001.

To investigate which exosomal miRNA was involved in EV-A71 infection, we isolated exosomes from the supernatants of uninfected and EV-A71 infected rhabdomyosarcoma (RD) cells. An ExoQuick-TC isolation kit was used for preliminary exosomes extraction. Then, CD63 immunomagnetic beads were used to eliminate free virus particle contamination and isolate pure, homogenous exosomes (Figure. 1A). The morphology of exosomes particles was confirmed by transmission electron microscopy (TEM) and nanoparticle tracking analysis (NTA) (Figure. S1A, S1B). Western blot (WB) analysis showed expression of exosomes markers CD63, tumor suppressor gene 101 protein (TSG101), and heat shock protein 70 (HSP70) with the absence of the endoplasmic reticulum marker calnexin (Figure. 1B). The expression of exosomal miRNA from EV-A71-infected and mock-infected cells was profiled with Agilent Human miRNA Microarray (data not shown). Based on this, quantitative real-time polymerase chain reaction (qRT-PCR) was applied to validate miRNA-155 expression. The results showed that EV-A71-infected RD cells expressed higher levels of endogenous miR-155 than mock-infected RD cells (Figure. 1C). qRT-PCR analysis (Figure. 1D) revealed that miR-155 was significantly more abundant in equivalent exosomes from infected cells (Exo-EV-A71) than from mock-infected cells (Exo-mock). Taken together, these findings demonstrate that exosomal miR-155 expression was up-regulated in response to EV-A71 infection.

Exosomes are novel mediators that can enfold miRNAs. Human neuroblastoma (SK-N-SH) cells, a type of nerve cell, were used to study EV-A71 neuropathogenesis. To investigate the functional role of exosomal miR-155 during EV-A71 infection, we isolated exosomes from media of RD cells that had been transiently transfected with miR-155-mimic and miR-mimic-NC (miR-mimic negative control) (Figure. S2A, S2B). qRT-PCR analysis was performed to detect miR-155 expression in equivalent exosomes from each group. As shown in Figure. S2C, miR-155 was significantly increased in exosomes from cell culture supernatants after transfecting with miR-155-mimic compared to miR-mimic-NC. Exosomes from cells transfected with miR-155-mimic (Exo-155-mimic) were incubated with SK-N-SH cells for 4 h, then the cells were infected with free EV-A71 virus. At 36 h post-infection (p.i.), the expression levels of viral genomic RNA and viral structural protein VP1 were determined by qRT-PCR and WB, respectively. EV-A71 RNA was potently decreased by Exo-155-mimic in a dose-dependent manner (Figure. 1E), and VP1 protein was also decreased in Exo-155-mimic group compared to the Exo-mimic-NC group (Figure. 1F, 1G). All results indicated that exosomal miR-155 inhibits EV-A71 infection in SK-N-SH cells.

### Direct transfer of miR-155 to recipient cells SK-N-SH via exosomes

**Figure 2:**
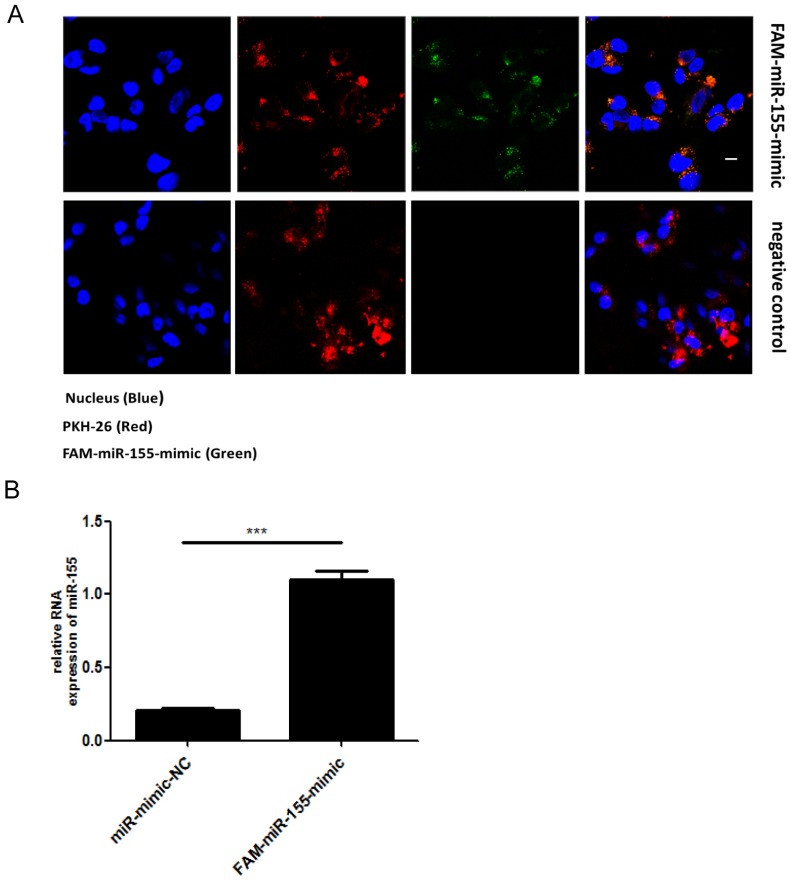
** Direct transfer of miR-155 to recipient cells SK-N-SH via exosomes. (A)** Confocal microscopy analysis of recipient SK-N-SH cells treated with purified PKH-26 labeled exosomes from RD cells transfected with FAM-miR-155-mimic or miR-mimic-NC. Green FAM signals, miR-155; red PKH-26 signals, exosomes; blue 4 , 6-diamidino-2-phenylindole (DAPI) signals, nuclei. Scale bar = 10 μm. **(B)** Expression of miR-155 in SK-N-SH cells incubated with exosomes from each group as measured by qRT-PCR. Data are from three independent experiments with each experiment performed in triplicate. ***p < 0.001.

Exosomes are extracellular vesicles found in various cells types that can deliver miRNAs to recipient cells. To determine whether miR-155 can be directly transferred to SK-N-SH cells via exosomes, we first isolated exosomes from RD cells that had been transiently transfected with fluorescein amidite (FAM) - miR-155-mimic. The exosomes derived from RD cells were labeled with a red fluorescent marker, PKH-26. Confocal microscopy analysis showed fluorescently labeled signals in the cytoplasm of recipient SK-N-SH cells incubated with exosomes from FAM-miR-155-mimic-transfected cells but not in those incubated with exosomes from mock-transfected cells (Figure. 2A). The presence of fluorescently labeled signals suggested that exosomal miR-155 can be internalized by the recipient SK-N-SH cells via exosomes. In addition, as shown in Figure. 2B, an increased abundance of miR-155 was detected in the recipient cells SK-N-SH treated with exosomal miR-155-mimic compared to those treated with exosomal miR-mimic-NC. These data indicate that exosomal miR-155 from RD cells was transferable to recipient SK-N-SH cells, increasing intracellular miR-155 expression in recipient cells.

### Exosomal miR-155 inhibits EV-A71 infection in SK-N-SH cells by targeting PICALM

Bioinformatics analysis (using TargetScans, miRanda, miRBase) showed that miR-155 can target the 3'UTR of PICALM at position 858-864 bp (Figure. S3A). The effect of miR-155 on a dual-luciferase reporter gene activity assay was abrogated when cells were transfected with the PICALM encoding sequence (Figure. S3B). To further confirm that PICALM is a direct downstream target of miR-155, we performed qRT-PCR and WB analysis of SK-N-SH cells transfected with miR-155-mimic or miR-mimic-NC to evaluate their effect on PICALM expression. After transfection with different concentrations of miR-155-mimic, whole cells were collected, and total RNA was extracted for qRT-PCR analysis. No significant decrease of PICALM mRNA level (Figure. S3C) was detected because miR-155 and the PICALM 3'UTR are not an exact match. Based on this result, we selected 50 nM as the optimum concentration of miR-155-mimic for WB analysis. Significantly, PICALM protein expression in cells transfected with miR-155-mimic was lower than in control (Figure. S3D). These results reveal that miR-155 suppresses PICALM protein expression through binding to PICALM's 3'UTR and PICALM is the gene target of miR-155 in SK-N-SH cells.

**Figure 3:**
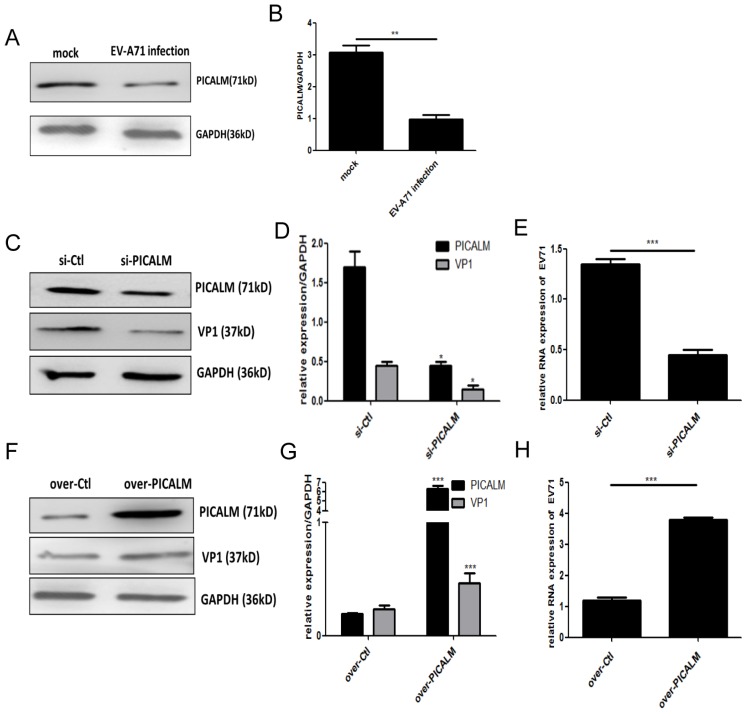
** PICALM is involved in EV-A71 infection of SK-N-SH cells. (A)** PICALM expression was determined during EV-A71 infection at 36 h p.i. **(C, F)** Si-PICALM, control interfering RNA (si-Ctrl), over-PICALM, and control overexpression vector (over-Ctrl) were used to transfect SK-N-SH cells. At 36 h p.i., cell lysates were prepared for detecting PICALM and VP1 protein expression. **(E, H)** EV-A71 genomic RNA levels in si-PICALM and over-PICALM transfected SK-N-SH cells were analyzed by qRT-PCR analysis. **(B, D, G)** Normalization of PICALM and VP1 protein with endogenous protein GAPDH was carried out with ImageJ software. *p < 0.05, **p < 0.01, ***p < 0.001. Data are from three independent experiments with each experiment performed in triplicate.

PICALM is a key component of clathrin-mediated endocytosis (CME) and participates in virus infection 11. We found that PICALM expression is down-regulated after EV-A71 infection (Figure. 3A, 3B), suggesting that this protein is involved in the infection. To investigate the regulatory function of PICALM during EV-A71 infection, PICALM interfering RNA (si-PICALM) was introduced into cells to knock down target gene expression in virus-infected cells. The reduction of PICALM resulted in down-regulated expression of VP1 protein and virus genomic RNA levels (Figure. 3C-3E). Conversely, expression of both the EV-A71 structural protein VP1 and virus genomic RNA was increased in cells transfected with a PICALM expression vector (over-PICALM) but not in those transfected with a negative control vector (over-Ctl) (Figure. 3F-3H). This is strong evidence that PICALM facilitates EV-A71 infection.

**Figure 4:**
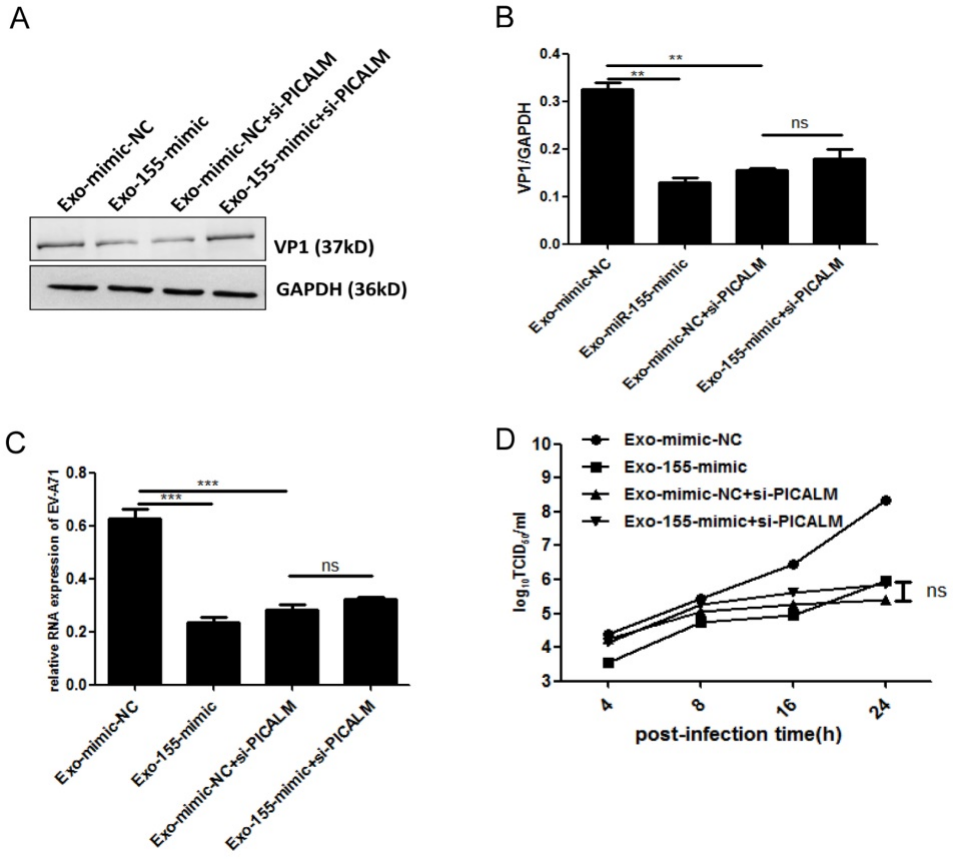
** Exosomal miR-155 inhibits EV-A71 infection in SK-N-SH cells by targeting PICALM. (A, C, D)** Viral protein, genomic RNA level, and virus titers were detected after different treatments including Exo-mimic-NC, Exo-155-mimic, Exo-mimic-NC + si-PICALM and Exo-155-mimic + si-PICALM during EV-A71 infection. **(A)** Cell lysate proteins were extracted and assessed by WB. **(B)** Normalization of VP1 protein with endogenous GAPDH was carried out with ImageJ software. **(C)** EV-A71 genomic RNA level were determined by qRT-PCR and normalized against endogenous GAPDH RNA level. **(D)** Virus titers in infected cells were determined at different time p.i. Data are representative of at least three independent experiments, each performed in triplicate. **p < 0.01, ***p < 0.001, ns, no significance.

To determine whether PICALM is required for exosomal miR-155-mediated inhibition of EV-A71 infection, si-PICALM was introduced into SK-N-SH cells that were subsequently treated with Exo-155-mimic or Exo-mimic-NC. Consistent with the above results, viral protein, genomic RNA and viral titers decreased significantly in cells transfected with Exo-155-mimic compared to Exo-mimic-NC (Figure. 4A). However, exosomal miR-155-mimic could not reduce the levels of viral protein, viral genomic RNA, or viral titers in PICALM knock-down cells compared to the exosomal miR-mimic-NC group (Figure. 4A-D). These results indicate that silencing PICALM blocks the inhibition of exosomal miR-155 during EV-A71 infection. We therefore concluded that exosomal miR-155 inhibits EV-A71 infection via a PICALM-dependent mechanism.

### Exosomal miR-155 inhibits EV-A71 infection *in vivo*

**Figure 5:**
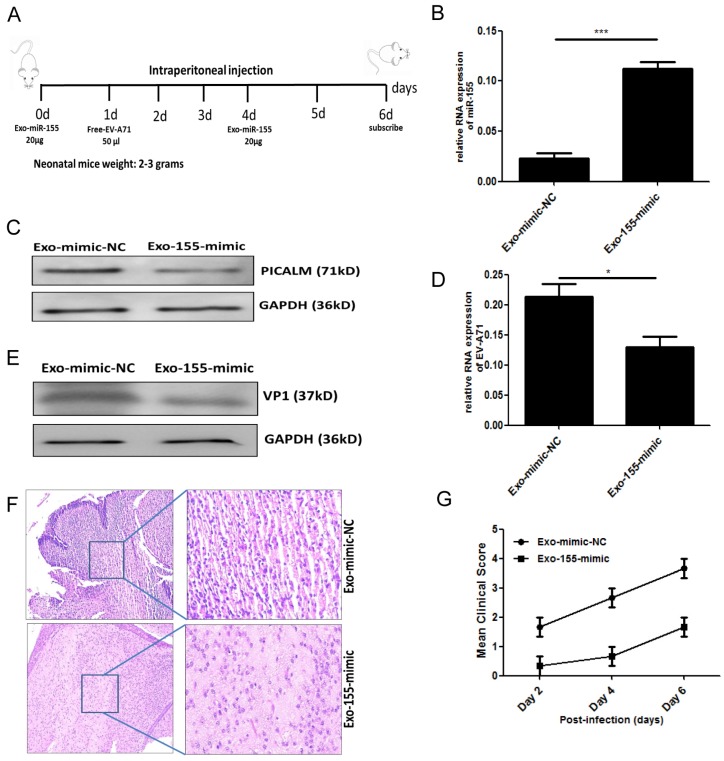
** Exosomal miR-155 inhibits EV-A71 replication *in vivo*. (A)** Exosomal miR-155-mimic (Exo-155-mimic) or exosomal miR-mimic-NC (Exo-mimic-NC) (20 µg) was administered to mice via intraperitoneal injection at two time points, day 0 and day 4. EV-A71 at a multiplicity of infection (MOI) of 1 (50 µl) was administered by intraperitoneal injection at day 1. **(B)** miR-155 levels in brain tissue were determined by qRT-PCR. **(C)** PICALM protein levels were determined by WB. **(D, E)** Viral RNA **(D)** and VP1 protein **(E)** levels after Exo-155-mimic treatment were determined by qRT-PCR and WB, respectively. **(F)** Hematoxylin and eosin (HE) staining was used to show the inflammatory response in brain tissue. **(G)** Six-day-old mice were observed, and their clinical symptoms and mean clinical score were recorded. Data are from three independent experiments, each performed in triplicate. *p < 0.05, ***p < 0.001.

To validate our *in vitro* findings, we investigated whether exosomal miR-155 would inhibit EV-A71 infection *in vivo*. Brain tissue from EV-A71-infected or mock-infected neonatal mice was isolated, and miR-155 expression was determined. A higher level of miR-155 was found in brain from EV-A71-infected mice (Figure. S4). Further *in vivo* experiments were performed by administering exosomal miR-155-mimic (Exo-155-mimic) or exosomal miR-mimic-NC (Exo-mimic-NC) to EV-A71-infected mice by intraperitoneal injection (Figure. 5A). PICALM expression was significantly down-regulated (Figure. 5C) with up-regulation of the miR-155 level (Figure. 5B). This result further validated the *in vitro* finding that the Exo-155-mimic directly targets PICALM. Moreover, we observed a significant decrease of viral genomic RNA (Figure. 5D) and VP1 expression (Figure. 5E) after Exo-155-mimic treatment. Interestingly, inflammatory cell infiltration in brain tissue was noticeably reduced in Exo-155-mimic mice compared to mice treated with Exo-mimic-NC (Figure. 5F). Based on clinical scoring criteria in EV-A71-infected neonatal mice 12, the clinical symptoms of the Exo-155-mimic group mice gradually disappeared and the mean clinical score concomitantly decreased compared to the Exo-mimic-NC group (Figure. 5G), suggesting that exosomal miR-155 can protect host cells from EV-A71 infection. These results strongly support the role of exosomal miR-155 in suppressing EV-A71 infection by targeting PICALM *in vivo*.

### High levels of miR-155 in throat swabs from EV-A71-infected patients

**Figure 6:**
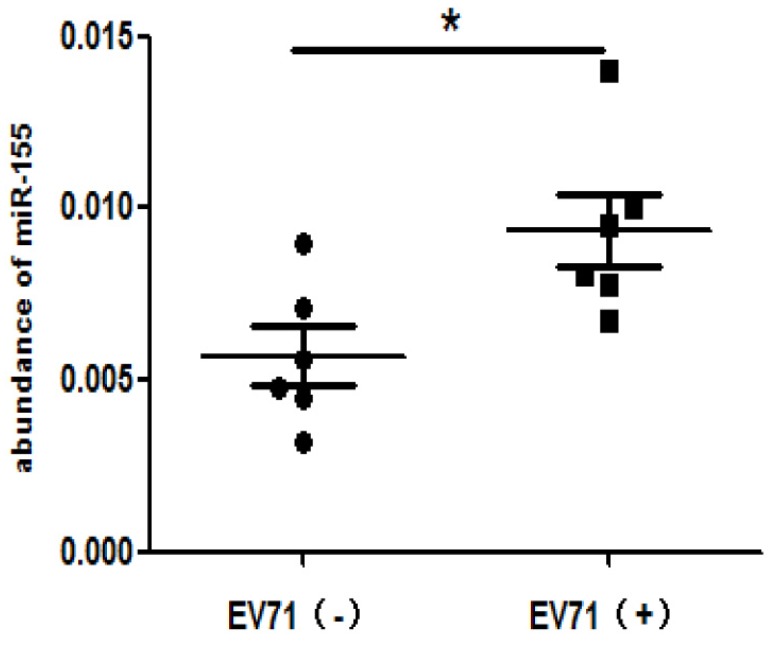
** High miR-155 levels in throat swabs from EV-A71-infected patients.** Total RNA was extracted from the throat swabs of infected (EV-A71 (+)) or uninfected (EV-A71 (-)) individuals, and miR-155 RNA levels were determined. U6 snRNA was used as an internal control. The symbols “+” and “-” indicate positive and negative samples, respectively. *p < 0.05.

To determine the clinical relevance of miR-155 in EV-A71-infected patients, miR-155 levels were measured in throat swabs from HFMD patients. Total RNA was extracted from the swab samples of infected and non-infected individuals. The miR-155 levels were higher in EV-A71-infected patients than in healthy individuals (Figure. 6), indicating that miR-155 levels in throat swabs are related to EV-A71 infection and may be involved in viral pathogenesis.

## Discussion

In this study, we demonstrated that exosomal miR-155 acts in host-pathogen interactions by repressing PICALM protein expression. During EV-A71 infection, host cells can respond by modifying miRNA expression to inhibit virus infection (e.g., miR-23b, miR-296-5p, miR-526a and miR-27a) 13-16. To investigate exosomal miR-155 in EV-A71 infection, we confirmed that miR-155 was highly expressed in exosomes derived from EV-A71-infected RD cells by miRNA chip and qRT-PCR analysis. Moreover, we presented evidence that exosomal miR-155 can be transferred to recipient cells and suppress viral infection by targeting PICALM.

MiR-155 is a well characterized miRNA associated with multiple biological processes and diseases. Bioinformatic analysis revealed at least 4174 targets of putative human miR-155 mRNA, with a total of 918 conserved sites 17. Thus, miR-155 affects multiple physiological and pathological functions, including those related to viral infection. Recent studies provided evidence that miR-155 is involved in antiviral innate immunity through Toll-like receptors (TLR3, TLR7, and TLR9) or the nuclear factor kappa-B (NF-кB) signaling pathway 18-20. The innate immune system provides an early phase defense to limit pathogen invasion by recognizing microbial moieties. Although TLRs and NF-кB signaling pathways play fundamental roles in host antiviral responses by inducing the expression of pro-inflammatory cytokines and type I interferons, many viruses also have different mechanisms to antagonize the innate immune response, helping to establish productive infections. To the host cell, it is particularly vital to provide antiviral strategies. Wang and colleagues determined that miR-155 acts as an anti-viral host factor, inhibiting infectious burse disease virus replication by targeting suppressor of cytokine signaling1 (SOCS1) and TRAF family member-associated NF-кB activator (TANK) 21. In some cases, viruses may regulate miR-155 to help in the viral life cycle and other aspects of pathogenesis. For instance, Epstein-Barr virus and HCV induce miR-155 to promote virus replication and infection 22, 23. Considerable experimental data has shown that the relationship between viruses and miR-155 is very complicated and worthy of further investigation.

Much of the recent increase of exosomes research focused on their roles that in transmitting signals and molecules via intercellular vesicle traffic. Exosomes secreted from various cells (e.g., tumor, immune, and nerve cell) have been shown to influence cell-to-cell communication 24. Many research groups demonstrated the critical role of exosomes in virus infection, such as for human immunodeficiency virus (HIV), hepatitis E virus, hepatitis B virus, and HCV 25-28. Roth and colleagues found exosomes from HIV-infected cells were enriched in specific miRNAs 29. Fu et *al*. discovered that exosomes secreted by EV-A71-infected cells selectively packaged high levels of miR-146a that could be transferred to recipient cells and facilitate EV-A71 replication 30. However, little is known about whether and how exosomal miR-155 modulates recipient cellular responses in EV-A71 infection.

We demonstrated that miR-155 levels were increased in exosomes from EV-A71-infected RD cells, and exosomal miR-155-mimic could inhibit EV-A71 infection *in vitro*. Unfortunately, there was no significant difference in the effect of the miR-155 inhibitor on EV-A71 (data not shown). There may be some compensatory pathway when miR-155 is knocked down during EV-A71 infection. To further validate the effects of exosomal miR-155 on EV-A71 infection *in vivo*, we established a mouse-adapted EV-A71 model 31 and introduced exosomal miR-155 through intraperitoneal injection. The *in vivo* miR-155 inhibition of EV-A71 replication was verified in brain tissue. Moreover, we detected miR-155 expression in throat swabs from EV-A71-infected individuals. The miR-155 expression level was higher in throat swabs from EV-A71-infected individuals compared to healthy ones. This observation indirectly supports the role of exosomal miR-155 in EV-A71 infection.

Some non-enveloped viruses have the capacity to directly penetrate the cytosol through the plasma membrane; most depend on CME, such as Adenoviridae and Reoviridae 32-34. As an important adaptor protein, PICALM is a key component of CME for viral invasion of host cells. It affects the susceptibility to viral infection in the brain 35 and is expressed in endothelial cells in human brain tissue 36, suggesting that PICALM may be involved in severe HFMD caused by EV-A71 infection. PICALM is also reportedly associated with the herpes simplex virus lifecycle, specifically involvement in viral entry and transport within the host cell 37, 38. We found that down-regulation of PICALM inhibited EV-A71 entry into SK-N-SH cells. Biological information analysis and subsequent studies demonstrated that exosomal miR-155 suppressed PICALM expression by binding to the PICALM 3'UTR, thus inhibiting viral infection. In summary, the present results show that PICALM is a direct target of miR-155 in recipient cells, and exosomal miR-155 inhibits EV-A71 infection by targeting PICALM.

In conclusion, our experiments in SK-N-SH cells and EV-A71-infected mice show that exosomal miR-155 acts as a virus inhibitor, suppressing EV-A71 infection through targeting PICALM *in vitro* and *in vivo*. Exosomal miR-155 may have a novel potential application as a therapeutic agent targeting clinical EV-A71 infection.

## Materials and Methods

### Cells, virus, and samples

RD and SK-N-SH cells were obtained from the cell bank of the Chinese Academy of Sciences. The EV-A71 strain was isolated from the throat swab of an EV-A71-infected patient and inoculated into RD cells for duplication. The isolated virus was confirmed using the Enterovirus A71 RNA Detection kit (S-SBIO, China).

Stool samples and throat swabs from HFMD patients were collected at The Affiliated People's Hospital, Jiangsu University, China. Viral Transport Medium (VTM) (Yocon, China) was used for the swab samples. Etiologic diagnosis was confirmed by detection of EV-A71 in stool samples using the Enterovirus A71 RNA Detection kit (S-SBIO, China). Ethical approval for the study was obtained from the hospital's ethics committee, and written informed consent was obtained from all participants prior to study enrollment.

### miRNA mimics and miRNA transfection

Chemosynthetic miR-155 oligonucleotides (miR-155-mimic), the miR-mimic-negative control (miR-mimic-NC), and the FAM-tagged miR-155 mimic (FAM-miR-155-mimic) were purchased from Ribo Biotech (Guangzhou, China). Lipofectamine 2000 (Thermo Fisher Scientific, USA) was used for miRNA transfection. Briefly, miRNAs and Lipofectamine 2000 were diluted in OPTI-MEM without fetal bovine serum (FBS), then mixed at room temperature for 20 min. Cells were incubated with the mixture in Dulbecco's modified Eagle's medium (DMEM) with no FBS at 37°C with 5% CO_2_. After 6 h of transfection, the culture media was replaced with fresh DMEM with 10% FBS.

MiR-155 targets were co-predicted by TargetScans (www.targetscan.org), miRanda (www.microrna.org/microrna/home.do), and miRBase (www.mirbase.org). The potential binding sites were identified as those with total sore ≥120; total energy <-17 kmol.

### Dual-luciferase reporter gene activity assay

A dual-luciferase reporter assay system kit (Promega, USA) was used to detect luciferase activity according to the manufacturer's protocol. In brief, plasmids (400 ng/well) were used to transfect HEK293T cells (4 × 10^5^ cells per well) in 24-well plates with Lipofectamine 2000, following the manufacturer's instructions. After 24 h, the cells were collected and washed once with 1× phosphate-buffered saline (PBS). Then 100 µl passive lysis buffer was added to lyse the cells. After 15 min, the cell suspension was collected using a detector tube. Luciferase Assay Reagent (LAR II) was added to 20 µl of cells suspension and mixed gently. Luciferase activity was analyzed using a Victor3 multilabel counter (PerkinElmer, USA).

### qRT-PCR

Total RNA was extracted from cultured cells or tissue samples using TRIzol reagent (Invitrogen, USA). Exosomes RNA was extracted using an RNAqueous-micro kit (Thermo Fisher Scientific, USA), according to the manufacturer's protocol. Briefly, for cellular mRNA analysis, 1000 ng of total RNA was used for reverse transcription with a PrimeScript RT reagent kit (Takara, Japan). qRT-PCR analysis was performed with Luminaris Color HiGreen qPCR Master Mix (Thermo Fisher Scientific, USA). GAPDH mRNA was used as an internal control for measuring cellular RNA expression. For miRNA analysis, 500 ng of total RNA was used for reverse transcription with 2× reverse transfection mix and 1 µl of cDNA was used for qRT-PCR with 2× SYBR Green mix. These two mixes were both from the Bulge-Loop miRNA qRT-PCR starter kit (Ribo Biotech, Guangzhou, China). U6 snRNA (for miRNA) and cel-miRNA-39 (for exosomal miRNA) were used as internal controls for quantifying miRNA expression. The primer sequences for EV-A71 (forward, 5ʹ-AGGATTTACATGAGAATGAAGCA-3ʹ, and reverse, 5ʹ-GCATAATTTGGGTTGGCTTT-3ʹ) were obtained from a published article 39.

### WB

Cells were collected and lysed using radioimmunoprecipitation assay buffer (Kangwei Century, China). Extracted proteins were analyzed by sodium dodecyl sulfate-polyacrylamide gel electrophoresis; then, transferred to polyvinylidene fluoride membranes (0.22 µm; BioRad, USA) as previously described 40. The membranes were then blocked in Tris-buffered saline containing Tween (TBST) containing 5% non-fat dried milk for 1 h. Then, the membranes were incubated with primary antibody overnight at 4°C. The following primary antibodies were used: anti- EV-A71 VP1 (a gift from Zhenjiang First People's Hospital, China), anti-PICALM (Novus, USA), anti-CD63 (Abcam, UK), anti-HSP70 (Abcam, UK), anti-TSG101 (Abcam, UK), and anti-calnexin (Abcam, UK), plus the internal control anti-GAPDH (Kangwei Century, China). The antibodies were diluted out according to the manufacturers' recommendations. The dilution for EV-A71 VP1 antibody was 1:1000. After three washes in TBST, membranes were incubated with secondary antibodies including goat anti-mouse IgG-HRP and goat anti-rabbit IgG-HRP (Kangwei Century, China) for 1 h at room temperature; then, they were washed a further three times. Finally, the proteins were visualized using enhanced chemiluminescence (ECL) substrate according to the manufacturer's protocol.

### Pre-isolation and purification of exosomes

Exosomes were prepared from cell supernatants with the following differential centrifugation: 300 *g* at 4°C for 20 min, 2000* g* at 4°C for 20 min, 10 000 *g* at 4°C for 30 min, followed by filtration through a 0.22-µm filter. Exosomes were then concentrated using a 100-kD ultrafiltration tube (Millipore, USA). ExoQuick-TC exosome isolation reagent (System Biosciences, USA) was used to precipitate exosomes by centrifugation at 1500 *g* at 4°C for 30 min. Exosomes were re-suspended in PBS and incubated with anti-CD63 antibody, followed by a corresponding secondary antibody coupled to magnetic beads (Miltenyi Biotec, Germany) to further purify the exosomes. The Miltenyi Biotec MidiMACS separator combined with LD columns (catalog no. 130-042-901) was used for exosome isolation.

### Characterization of exosomes

TEM, WB, and NTA were used for exosome characterization. Briefly, for TEM detection, exosomes suspended in 200 µl of PBS were fixed with 4% paraformaldehyde and 4% glutaraldehyde in 0.1 M phosphate buffer (pH 7.4) and kept at 4°C until needed. Each exosome sample was placed on a carbon-coated copper grid, immersed in 2% phosphotungstic acid solution (pH 7.0) for 30 s, and then examined with a transmission electron microscope (JEM-1200EX; JEOL Ltd., Japan) at an acceleration voltage of 80 kV. WB analysis used the following primary antibodies: anti-CD63, anti-HSP70, anti-TSG101, and anti-calnexin. The proteins were visualized with the Clarity Western ECL substrate chemiluminescence system (BioRad, USA), according to the manufacturer's protocol, using the Fujifilm LAS-4000 luminescent image analyzer. For NTA analysis, the NanoSight LM10 system equipped with fast video capture and particle-tracking software (NanoSight, UK), was used to analyze the exosomes. Data analysis was performed with NTA 2.3 software (NanoSight, UK) 41-42.

### Quantification of exosomes

Exosomal concentration is expressed as the total immunoreactive exosomal CD63 (ExoELISA-ULTRA CD63, System Biosciences, USA). In brief, 50 µl of exosome protein samples were immobilized in microtiter plate wells and incubated at 37°C for 1 h. The plate was washed using wash buffer three times for 5 min each. Then, 50 µl of an exosome-specific primary antibody (anti- CD63) was added to each well and incubated at room temperature for 1 h with shaking. Plate was washed using wash buffer, and 50 µl secondary antibody was added to each well before the plate was incubated at room temperature for 1 h with shaking. Then, the plate was washed using wash buffer and 50 µl of super-sensitive 3,3',5,5'-tetramethylbenzidine enzyme-linked immunosorbent assay substrate was added, and the plate incubated at room temperature for 15 min with shaking. Finally, 50 µl of stop buffer was added to terminate the reaction and provide a fixed endpoint for the assay. Absorbance was measured at 450 nm. The number of exosomes/ml was obtained using an exosomal CD63 standard curve.

### Labeling and uptake of exosomes

Exosomes containing FAM-miR-155-mimic were labeled using the PKH-26 Red Fluorescent Cell Linker kit (Sigma, USA). This suspension was then incubated at 37°C for 20 min. Excess dye was removed using a 100-kD ultrafiltration tube (Millipore, USA) at 1500 *g* for 30 min. The labeled exosomes were incubated with SK-N-SH cells for 4 h. Exosome uptake by the recipient cells was observed using a Leica TCS SP5 II laser scanning confocal microscope (Leica, Germany).

### Construction of an EV-A71mouse infection model

One-day-old ICR neonatal mice were inoculated with a 10^6^ 50% tissue culture infective dose of virus by intraperitoneal injection. Exosomal miR-155-mimic and exosomal miR-mimic-NC were also introduced into neonatal mice by intraperitoneal injection. The mice were sacrificed at 7 days to obtain brain tissue for further experiments. The animal experiments were approved by the institutional animal care and use committee.

### Statistical analysis

All graphs and statistical analyses were performed using GraphPad Prism ver. 5 (GraphPad Software Inc., USA). All statistics were based on at least three independent experiments. Data were analyzed using one-way analysis of variance for comparisons among groups. Results are expressed as means ± standard error of the mean (SEM). In this study, *p*-values < 0.05 were considered significant, and two-tailed tests were employed.

## Supplementary Material

Supplementary figures and tables.

## Abbreviations

EV-A71: enterovirus A71
HFMD: hand, foot, and mouth disease
miRNA: microRNA
miR-155: microRNA-155
PICALM: phosphatidylinositol clathrin assembly protein
3ʹUTR: 3ʹ untranslated region
HCV: hepatitis C virus
RD cells: rhabdomyosarcoma cells
WB: western blot
TEM: transmission electron microscopy
NTA: nanoparticle tracking analysis
qRT-PCR: quantitative reverse-transcription polymerase chain reaction
Exo-EV-A71: exosomes from infected cells
SK-N-SH cells: human neuroblastoma cells
miR-mimic-NC: miR-mimic negative control
VP1 protein: viral structural protein 1
CD63: tetraspanin 63
HSP70: heat shock protein 70
TSG101: tumor suppressor gene 101 protein
calnexin: endoplasmic reticulum marker
U6snRNA: U6 small nuclear RNA
p.i.: post-infection
FAM: fluorescein amidite
DAPI: 4ʹ,6-diamidino-2-phenylindole
CME: clathrin-mediated endocytosis
si-PICALM: PICALM interfering RNA
over-PICALM: PICALM overexpression vector
si-Ctrl: control interfering RNA
over-Ctrl: control overexpression vector
MOI: multiplicity of infection
TLR3: Toll-like receptor 3
NF-кB: nuclear factor kappa-B
FBS: fetal bovine serum
DMEM: Dulbecco's modified Eagle's medium
PBS: phosphate-buffered saline
LAR II: Luciferase Assay Reagent
GAPDH: glyceraldehyde 3-phosphate hydrogenase
TBST: Tris-buffered saline containing Tween
ECL: enhanced chemiluminescence
