# Supplementary material for: Exosomal MicroRNA-155 Inhibits Enterovirus A71 Infection by Targeting PICALM
Source: Int J Biol Sci. 2019 Nov 15;15(13):2925–35. doi: 10.7150/ijbs.36388 (PMC6909958; doi:10.7150/ijbs.36388)
Supplement: Supplementary file 1 — Supplementary figures and tables. [file ijbsv15p2925s1.pdf]

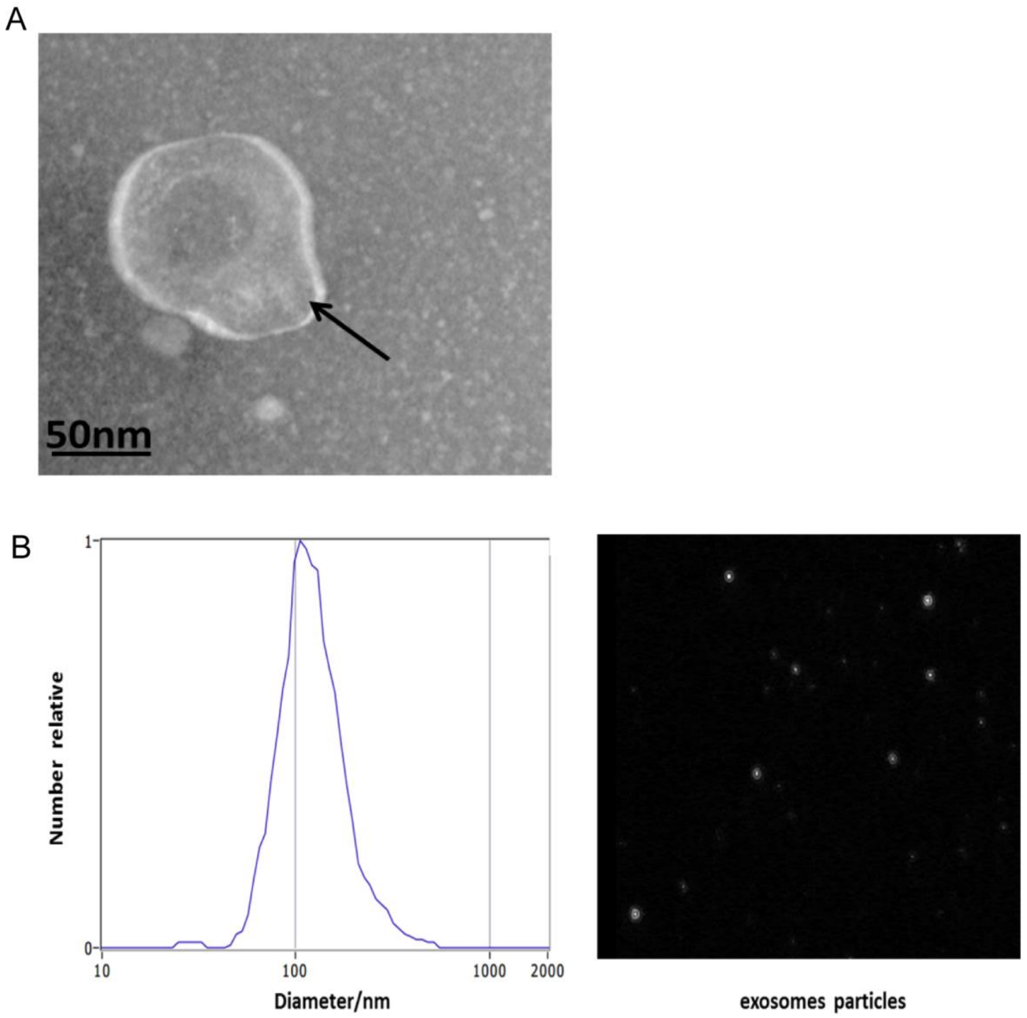

Figure S1

Figure S1. The purification and identification of exosomes. (A) The identification of $CD63^+$ exosomes by TEM (scale bar = 50 nm). (B) NTA showed the diameter of exosomes is approximate 110 nm (left chart) and exosomes particles are homogeneous (right chart).

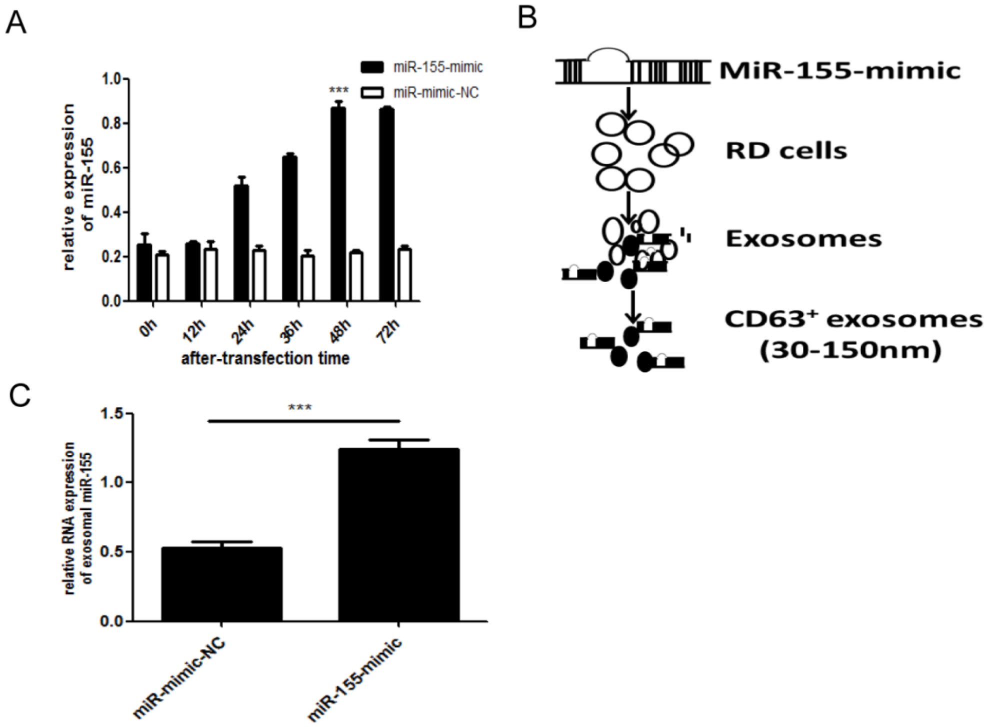

Figure S2

Figure S2 (A) miR-155-mimic and miR-mimic-NC were transfected into cells and levels of miR-155 was detected by qRT-PCR analysis at different transfection times. U6snRNA was used for normalization of miR-155 expression. (B) $CD63^+$ exosomes were isolated and collected according to the flow chart. (C) Total RNA was extracted from exosomes and miR-155 levels were detected by qRT-PCR analysis. Data are from three dependent experiments, each performed in triplicate. ***, $p < 0.001$.

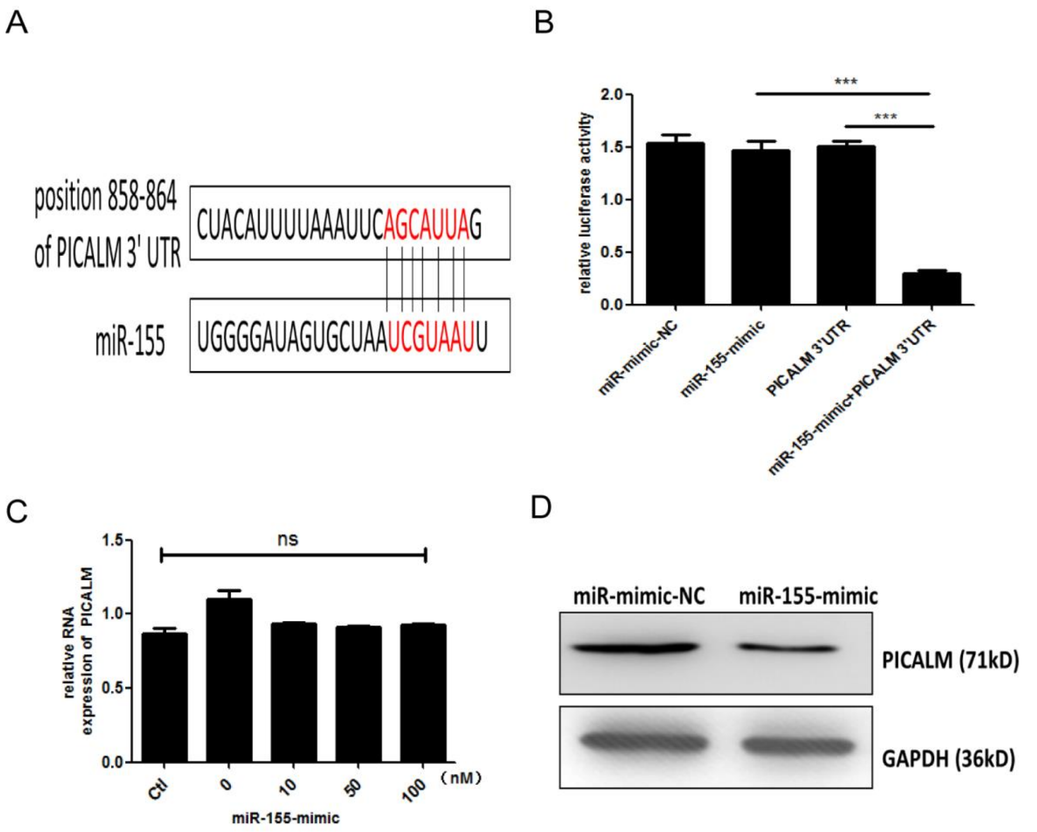

Figure S3

Figure S3. PICALM is the target of miR-155 in SK-N-SH cells. (A) A consensus miR-155 binding site was identified within the un-coded sequence of PICALM. (B) Co-transfection of miR-155-mimic and luciferase vector with PICALM un-coded sequences decreased the luciferase activities in HEK-293T cells. A 50 nmol/L final concentration of miR-155-mimic was added in SK-N-SH cells. (C, D) The RNA and protein level of PICALM were detected respectively by qRT-PCR and WB analysis. Data are from three dependent experiments, each performed in triplicate.***, $p < 0.01$; ns, no significance.

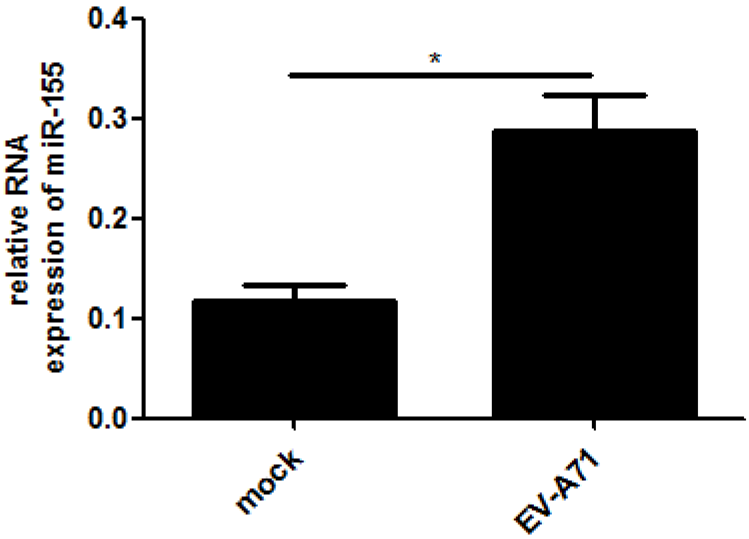

Figure S4

Figure S4. MiR-155 is up-regulated in EV-A71 infected mice. A qRT-PCR analysis of miR-155 levles in brain tissue from EV-A71 infected neonatal mice. U6snRNA was used for normalization of miR-155 levles. Data are from three dependent experiments, each performed in triplicate.*, p < 0.05.
